# Alternative biomarkers of tuberculosis infection in patients with immune-mediated inflammatory diseases

**DOI:** 10.3389/fmed.2023.1271632

**Published:** 2023-11-23

**Authors:** Elisa Petruccioli, Linda Petrone, Saeid Najafi-Fard, Assunta Navarra, Valentina Vanini, Gilda Cuzzi, Fabrizio Cantini, Gina Gualano, Fabrizio Palmieri, Delia Goletti

**Affiliations:** ^1^Translational Research Unit, National Institute for Infectious Diseases Lazzaro Spallanzani-IRCCS, Rome, Italy; ^2^Department of Epidemiology, National Institute for Infectious Diseases Lazzaro Spallanzani-IRCCS, Rome, Italy; ^3^Rheumatologist Consultant, Private Practice, Siena, Italy; ^4^Respiratory Unit, National Institute for Infectious Diseases Lazzaro Spallanzani-IRCCS, Rome, Italy

**Keywords:** IGRA, IFN-γ, IP-10, tuberculosis infection, diagnosis, latency

## Abstract

**Introduction:**

IFN-γ release assays (IGRAs) are one of the referral tests for diagnosing tuberculosis infection (TBI). To improve IGRAs accuracy, several markers have been investigated. Patients with immune-mediated inflammatory diseases (IMID), taking biological drugs, have a higher risk to progress to TB-disease compared to the general population. In several guidelines, annual TBI screening is recommended for patients undergoing biological therapy. Aim of this study was to investigate, within the QuantiFERON-TB-Plus (QFT-Plus) platform, if beside IFN-γ, alternative biomarkers help to diagnose TBI-IMID patients.

**Methods:**

We enrolled 146 subjects: 46 with TB disease, 20 HD, 35 with TBI and 45 with TBI and IMID. Thirteen IMID subjects with a QFT-Plus negative result were diagnosed as TBI based on radiological evidence of TBI. We evaluated the IP-10 level in response to TB1 and TB2 peptides of QFT-Plus assay and we compared these results with the standardized assay based on IFN-γ. Multiplex immune assay was performed on plasma from TB1 and TB2 tubes and results were analyzed by a gradient boosting machine (GBM) as learning technique.

**Results:**

TBI-IMID showed a significant decreased IP-10 level in response to TB1 and TB2 stimulation compared to TBI-NO IMID (*p* < 0.0001 and *p* = 0.0002). The TBI-IMID showed a moderate agreement between the IP-10-based assay and QFT-Plus scores. In TBI-IMID, QFT-Plus showed 70% sensitivity for TBI detection whereas the IP-10-based assay reached 61%. Tests combination increased the sensitivity for TBI diagnosis up to 77%. By a GBM, we explored alternative biomarkers for diagnosing TBI in IMID population reaching 89% sensitivity. In particular, the signature based on IL-2, IP-10, and IL-9 detection was associated with TB status (infection/disease). However, by applying the cut-off identified by ROC analysis, comparing TB and TBI with the HD group, within the IMID population, we did not improve the accuracy for TBI-diagnosis. Similarly, this signature did not improve TBI diagnosis in IMID with radiological evidence of TBI but negative QFT-Plus score.

**Discussion:**

To develop alternative strategies for TBI immune-diagnosis, future studies are needed to evaluate the memory response of TBI defined by radiological tools. These results may help in tuberculosis management of patients taking lifelong immune-suppressive drugs.

## Introduction

Tuberculosis (TB), caused by *Mycobacterium tuberculosis* (Mtb), is the second top cause of death by an infectious disease worldwide (1) after COVID-19.

Conventionally, TB infection was named “latent tuberculosis infection” (LTBI). Recently, it has been deeply demonstrated that the spectrum of conditions from the Mtb latency state to the TB disease state is much more complex, including a “continuum” of conditions ranging from uninfected individuals, TB infection, incipient TB, subclinical TB, and TB disease. Therefore, the WHO has strongly suggested to use the term TB infection (TBI) instead of LTBI (2–5).

Commonly, TBI is diagnosed by skin tests or Interferon (IFN)-γ release assays (IGRA), which are immune assays based on IFN-γ production after stimulation with Mtb-specific antigens (6–11).

WHO TB targets include TB incidence reduction by 90% and deaths by 95% within the year 2035 (1). Therefore, the diagnosis of TBI subjects and the administration of TB preventive treatment are critical steps to achieve the End TB Strategy targets (12). Individuals with an impaired immune response such as people living with HIV (13–15) or treated with specific drugs acting on the immune system (16–19) have a higher risk of developing TB disease. This fragile population includes subjects with immune-mediated inflammatory diseases (IMID), such as rheumatic diseases (RD) and/or dermatological diseases (DD). Glucocorticoids and/or methotrexate (MTX) are immunosuppressive drugs commonly used for IMID treatment; second-line treatment is represented by biological agents targeting different actors of the immune response such as tumor necrosis factor (TNF)-α, interleukin (IL)-1, IL-6, IL-12/IL-23, and IL-17, lymphocyte expressing CD20 or CD28. Patients with rheumatic disorders taking TNF inhibitors have the highest risk of developing TB disease than the general population (16–19). Some guidelines or practices recommend annual screening for TBI in patients undergoing biologic therapy (20). Hence, proper and rapid diagnosis and treatment of these patients at higher risk for TB reactivation are of the utmost importance (21).

Studies on serial testing of healthcare workers highlighted a frequent inconsistency of the QuantiFERON platform including QuantiFERON-TB-Plus (QFT-Plus) quantitative results ranging above the cut-off value (≥ 0.35 IU/mL) with possible reversions or conversion (22–26). Based on this evidence, it has been proposed a “border zone” of QFT-Plus ranging from a lower limit of 0.15–0.20 to an upper limit of 0.70–0.80 (25, 27–31). Although it has been proposed that an algorithm to improve the consistency of serial QuantiFERON-TB testing reduced the technical assay variability (28), persons with comorbidities such as IMID have a higher risk of having false-negative QFT-Plus results (25) or results falling within an uncertain range (32). In fact, although the TBI-IMID mount an Mtb-specific CD4 response producing IFN-γ, TNF-α, and IL-2 (33), they are characterized by a low IFN-γ response to QFT-Plus stimulation (32). For these reasons, the TBI screening of IMID subjects may be challenging, even more if not associated with TB contact screening but with a routine screening for properly assessing the feasibility of IMID biological therapy. False-negative QFT-Plus results in IMID subjects may result in TB reactivation due to a lack of TB preventive therapy administration, usually proposed before starting the immune-suppressive IMID treatment in those scored with TBI at screening (30).

To improve the accuracy of the IGRAs, several markers, different from IFN-γ, have been investigated (6, 34). In particular, IFN-γ-induced protein 10 (IP-10) has been deeply and largely studied (35–45) in patients with IMID (46). IP-10 is a chemokine secreted by antigen-presenting cells and induced by several cytokines, especially by IFN-γ. Several findings propose IP-10 as a promising biomarker of TB infection (6, 31, 42, 47–51). IP-10 is a good TB biomarker in immunocompromised subjects such as people living with HIV (52, 53) or taking corticosteroids (54). Moreover, *in vitro* studies demonstrated that dexamethasone did not significantly affect IP-10 production (55) and the combination of the positive scores to IGRAs and IP-10-based tests increases the number of TBI diagnoses in rheumatic patients (46). As the IP-10 level is 100-fold higher compared with IFN-γ (46, 56), it is suitable to be explored for developing simplified and miniaturized assays, such as lateral flow, dried blood spots, and molecular detection (39, 43, 46, 57, 58).

The aim of this study was to investigate within the QFT-Plus platform if IP-10 compared with IFN-γ is a good biomarker for TBI in subjects with IMID under different immunosuppressive drug regimens. Moreover, we evaluated a large cytokine profile in response to QFT-Plus stimulation with the aim of finding new biomarkers of TBI.

## Methods

### Study participants

HIV-uninfected patients with TB disease, TBI, IMID, and healthy subjects were prospectively enrolled from October 2015 until March 2021. The Ethical Committee of “L. Spallanzani” National Institute of Infectious Diseases (INMI) approved the study (approval number 72/15 and 27/2019). Written informed consent was required to participate in the study. Part of the patients' samples were used in a published study (49).

As the IMID status in those with TBI is associated with a higher risk of progressing to TB disease (23), IMID patients with TBI with either rheumatic diseases (RDs) or dermatological diseases (DDs) undergo preventive therapy before starting biological therapy. As controls, we included TBI-NO IMID patients who were evaluated as contacts of patients with TB disease or screened for study requirements or because originated from a high TB-endemic country. The diagnosis of TBI was based on a positive score to QFT-Plus (Diasorin, Vicenza Italy) and the absence of clinical, microbiological, and radiological evidence of TB disease. IMID patients with a negative QFT-Plus but with radiological evidence of scars in the upper lung lobes and reported past exposure to TB cases were considered TBI and preventive therapy was proposed; one of the enrolled patients developed TB disease during the time of observation (at least 6 months after the enrolment).

Patients with TB disease were defined based on a “microbiological diagnosis” (positive Mtb culture) or by a “clinical diagnosis” (clinical and radiologic criteria, exclusion of other diseases, and a good response to TB therapy). Patients with TB disease were enrolled within 7 days of starting TB therapy. Demographic and clinical data for TB patients and healthy donors were collected and are shown in Table 1.

**Table 1:** Demographic and clinical characteristics of the enrolled patients employed to perform the ELISA and LUMINEX studies.

	***N* (%)**	**TB**	**HD**	**TBI-NO IMID**	**TBI-IMID**	**Total**	***p***
		**46**	**20**	**35**	**45**	**146**	
	Median age N (IQR)	37 (30–44.25)	44.5 (33.25–51)	44 (32–55)	59 (48–69.5)		< 0.0001^#^
	Male gender *N* (%)	24 (52.0)	8 (40)	10 (28.5)	18 (40)	60 (41)	0.2008^*^
Origin *N* (%)	Western Europe	18 (39)	20 (100)	20 (57.1)	31 (69)	89 (61)	na
	Eastern	14 (30)	–	6 (17.1)	8 (18)	28 (19)
	Asia	5 (11)	–	1 (3)	2 (4)	8 (5.5)
	Africa	5 (11)	–	4 (11.4)	1 (2)	10 (7)
	South America	4 (9)	–	4 (11.4)	3 (7)	11 (7.5)
TB diagnosis	Clinical	12 (26)	–	-	-	12 (26)	-
	Microbiological	34 (74)	–	-	-	34 (74)	
BCG *N* (%)	Vaccinated	27 (59)	0 (0)	14 (40)	14 (31)	55 (38)	< 0.0001^*^
	Unvaccinated	19 (41)	20 (100)	21 (60)	31 (69)	91 (62)	
X-ray findings of TBI^**^	Positive	/	/	5 (24)	21 (54)	26 (43)	0.0313
	Negative			16 (76)	18 (46)	34 (57)	

TB, tuberculosis; TBI, tuberculosis infection; HD, Healthy donors; IMID, inflammatory mediated immune disease; N, number; IQR, interquartile range; BCG, Bacillus Calmette-Guérin.

# Kruskall-Wallis test.

* Chi-square test. na, Chi square test is not available because the Chi calculations are only valid when all expected values are greater than 1.0 and at least 20% of the expected values are greater than 5; these conditions have not been met, and thus the chi-square calculations are not valid;

** X-ray findings of TBI = radiological findings, such as fibrosis and/or calcification, have been reported for TBI-IMID subjects, percentages have been calculated on subjects with available chest-X-ray (beside having the radiological reports). The radiological images were not available for 14 TBI-NO IMID scored positive for QFT-Plus and 6 TBI-IMID scored positive for QFT-Plus.

To perform this study, we followed the STROBE statement checklist for case–control studies (https://www.strobe-statement.org/fileadmin/Strobe/uploads/checklists/STROBE_checklist_v4_case-control.pdf).

### QFT-plus and IP-10 detection

QFT-Plus (Diasorin) and IP-10 (Bio-Techne, Minneapolis, USA) assays were performed on the plasma of 122 subjects (36 TB, 26 TBI, 44 TBI-IMID, and 16 HD); 1 ml of blood was drawn into each QFT-Plus tube (Qiagen, Hilden, Germany): Nil-tube for the unstimulated condition, TB1-tube containing Mtb-specific peptides, TB2-tube containing Mtb-specific peptides, and mitogen-tube as positive control. Plasma supernatants were collected, and aliquots of supernatants were stored at −80°C until use.

IFN-γ was measured in QFT-Plus supernatants by ELISA. The assay was performed according to the manufacturer's instructions (59). The results were analyzed by QFT-Plus Analysis Software (www.quantiFERON.com) and evaluated according to the manufacturer's criteria. All patient samples were positive for mitogen.

IP-10 level was measured in QFT-Plus supernatants using Human CXCL10/IP-10 Quantikine ELISA (R&D Systems, Abingdon, UK) according to the manufacturer's instructions. The samples were tested as diluted 1:50. The concentration range of detection was 7.8–500 pg/mL.

### Multiplex analysis

Multiplex immune assay was performed on plasma harvested from TB1 and TB2 tubes. We used Bio-Plex Pro Human Cytokine 27-plex Assay panel and the MagPix system (all from Bio-Rad, Hercules, CA, USA), according to manufacturer's instructions to evaluate cytokines, chemokines, and growth factors [IL-1β, IL-1RA, IL-2, IL-4, IL-5, IL-6, IL-7, IL-8, IL-9, IL-10, IL-12p70, IL-13, IL-15, IL-17A, eotaxin, basic fibroblast growth factor (FGF), granulocyte colony-stimulating factor (G-CSF), granulocyte–macrophage colony-stimulating factor (GM-CSF), IFN-γ, IP-10, monocyte chemoattractant protein-1 (MCP-1), macrophage inflammatory protein (MIP)-1α, MIP-1β, platelet-derived growth factor (PDGF), RANTES (regulated on activation, normal T cell expressed and secreted), tumor necrosis factor-alpha (TNF)-α, and vascular endothelial growth factor (VEGF)]. Bio-Plex Manager software was used to generate raw data. All the values below the detection range were set as zero, and values above the detection range were converted to the highest value of the standard curve. Values of the unstimulated controls were subtracted from each condition. Samples with acquired bead count < 50 were excluded from the final analysis.

### Statistical analysis

Data were analyzed using Graph Pad (GraphPad Prism 8 XML ProjecT), Stata (StataCorp. 2021. Stata Statistical Software: Release 17. College Station, TX: StataCorp LLC), and R Project Software (version 4.2.1). Medians and interquartile ranges (IQRs) were calculated. Mann–Whitney U test for comparisons among groups; chi-squared test for categorical variables; receiver operating characteristic (ROC) analysis for evaluating the area under the curve (AUC) and the diagnostic performance; Spearman's rank correlation to measure the strength of association between two variables and the direction of the relationship (positive or negative). Test concordance was assessed by k-statistics where k ≤ 0.20 was considered “slight,” 0.20 < k ≤ 0.40 was considered “fair,” 0.40 < k ≤ 0.60 was considered “moderate,” 0.60 < k ≤ 0.80 was considered “substantial,” and 0.80 < k ≤ 1.00 was considered “optimal”. A two-tailed *p-*value < 0.05 was considered statistically significant.

The gradient boosting machine (GBM) technique, a supervised machine learning method, was applied to the Luminex data set to classify subjects according to their TB status. Due to the small number of subjects available, the classification model was built with leave-one-out cross-validation as a resampling method. The final parameter value sets for the model were as follows: iterations=50, the complexity of the tree= 3, learning rate = 0.1, and the minimum number of training set samples in a node to commence splitting = 10.

To investigate the association of the increasing concentration of analytes with any clinical status, a multinomial logistic regression model was applied to the Luminex data set; the association was expressed as a relative risk ratio (RRR) for a 10-unit increase.

## Results

### Demographic and clinical characteristics of the study population

We enrolled 146 subjects: 46 with TB disease, 20 with HD, 35 with TBI, and 45 with TBI and IMID; 34 TB disease patients underwent a microbiological diagnosis, whereas 12 underwent a clinical diagnosis. No differences were found in terms of sex; differently, the median age was higher in TBI-IMID subjects (*p* < 0.0001) than in other groups. Most of the enrolled subjects came from Western European countries and were BCG-unvaccinated (*p* < 0.0001) (Table 1). Thirteen IMID subjects with a QFT-Plus negative result were diagnosed as TBI based on radiological signs suggestive of TBI (60) (Table 1).

### Characteristics of the TBI-IMID group

Among the IMID subjects, 13 were negative on QFT-Plus and tuberculin skin test (TST). TBI diagnosis was based on chest X-ray findings at the apical level of the lungs and the history of TB exposure; TB disease was excluded based on the absence of clinical and microbiological signs of disease (60). Based on the evidence of a high risk of progressing to TB disease in IMID-TBI patients undergoing biological therapy (12, 15), TB preventive therapy was proposed: 31% of TBI-IMID patients were under immune-suppressive drugs in combination with NSAIDs or corticosteroids and 31% were not taking any treatment (Table 2). The TBI-IMID were stratified according to the type of disease in two main groups (Table 2): the group of “rheumatoid arthritis,” including rheumatic diseases such as rheumatoid arthritis, psoriatic arthritis, ankylosing spondyloarthritis, and polymyalgia rheumatic, and the group of “inflammatory skin disease.” Most of the TBI-IMID belonged to the “rheumatoid arthritis” group (89%); among them, 30% were under immune-suppressive drugs in combination with NSAIDs or corticosteroids and 30% did not undergo any treatment. Within the “inflammatory skin disease” group, 40% of the patients were taking immune-suppressive drugs in combination with other drugs and 40% were not taking any treatment (Table 2). All the TBI-IMID patients with a negative QFT-Plus score belonged to the “rheumatoid arthritis” group, and 46% of them were not taking any treatment at enrolment (Table 3). Regarding the other patients enrolled, therapy did not have an impact on the QFT-Plus score (*p* = 0.25, Table 3). Moreover, IMID therapy did not have an impact on IFN-γ production in response to QFT-Plus (Supplementary Figures 1A, B).

**Table 2:** Stratification of the patients based on the ongoing IMID therapy among the TBI-IMID patients enrolled.

		**IMID therapy** ***N*** **(%)**						**Type of therapy combination** ***N*** **(%)**					
**TBI-IMID**	***N*** **(%)**	**Immune-suppressive drugs**	**Corticosteroids**	**NSAIDs**	**Biological treatment**	**No therapy**	**GROUP** ***N*** **(%)**	**Biological treatment** + **other drugs**	**Immune-suppressive drugs** + **other drugs (no-Biologic)**	**Only biological treatment**	**One drug**	**No therapy**	*p* ^§^
Total	45	19 (42)	12 (27)	14 (31)	7 (15)	14 (31)		3 (7)	14 (31)	4 (9)	10 (22)	14 (31)	0.037
Rheumatoid arthritis	21 (47)	9 (47.3)	6 (50)	4 (28.6)	3 (43)	7 (50)	Rheumatic disease 40 (89)	3 (7.5)	12 (30)	4 (10)	9 (22.5)	12 (30)	NA
Psoriatic arthritis	11 (24.4)	6 (31.5)	4 (33)	5 (36)	1 (14)	3 (21)							
Ankylosing spondyloarthritis	6 (13.3)	1 (5.3)	0 (0)	3 (21.4)	2 (29)	2 (14)							
Polymyalgia rheumatica	1 (2)	1 (5.3)	1 (8)	0 (0)	0 (0)	0 (0)							
Systemic lupus erythematosus	1 (2)	0 (0)	0 (0)	0 (0)	1 (14)	0 (0)							
Chronic inflammatory skin diseases	5 (11)	2 (10)	1 (8)	2 (14)	0 (0)	2 (14)	Inflammatory skin disease 5 (11)	0 (0)	2 (40)	0 (0)	1 (20)	2 (40)	NA

TBI, tuberculosis infection; IMID, inflammatory-mediated immune disease; N, number; NSAIDS, non-steroidal anti-inflammatory drugs, type of biologics: 4 patients under anti-TNF-α, 1 patient under Janus kinase (JAK) Inhibitors; 2 patients under anti-IL-6 receptor;

§ Chi-square test, NA, Not Available because Chi calculations are only valid when all expected values are greater than 1.0 and at least 20% of the expected values are greater than 5; these conditions have not been met, and thus, the chi-square calculations are not valid.

**Table 3:** QFT-Plus response stratified based on the therapy and type of IMID in TBI-IMID patients.

		**Type of therapy** ***N*** **(%)**					**Type of therapy combination** ***N*** **(%)**					**Type of IMID** ***N*** **(%)**		
**TBI-IMID**	***N***	**Immune-suppressive drugs**	**Corticosteroids**	**NSAIDs**	**Biological treatment**	**No therapy**	**Combination of drugs** ^##^	**Only Biological treatment**	**One drug**	**No therapy**	**p** ^#^	**Rheumatic disease**	**Inflammatory skin disease** ^##^	**p** ^§^
QFT-plus positive	31	15 (48)	10 (3)	12 (39)	5 (16)^*^	7 (22)	14 (45)	2 (6)	8 (26)	7 (22)	0.25	27 (87)	4 (13)	0.3
QFT-plus negative	13	4 (30)	2 (15)	2 (15)	2 (15) ^**^	6 (46)	3 (23)	2 (15)	2 (15)	6 (46)	13 (100)	0 (0)		
QFT-plus positive/IP-10-based assay negative	7	3 (43)	3 (43)	1 (14)	1 (14)^***^	2 (29)	4 (57)	0 (0)	1 (14)	2 (29)	NA	7 (100)	0 (0)	NA
QFT-plus negative/IP-10-based assay positive	3	1 (33)	1 (33)	0 (0)	0 (0)	2 (67)	1 (33)	0 (0)	0 (0)	2 (67)	3 (100)	0 (0)		

TBI, tuberculosis infection; IMID, inflammatory-mediated immune disease; N, number; NSAIDS, non-steroidal anti-inflammatory drugs;

* 4 patients under anti-TNF-α, 1 patient under Janus kinase (JAK) Inhibitors,

** 2 patients under anti-IL-6 receptor;

*** 1 patient under anti-TNF-α;

§ Fisher's exact test,

# chi-square test;

## it includes biological treatment + other drugs and immune-suppressive drugs + other drugs; NA, not available because the chi calculations are only valid when all expected values are >1.0 and at least 20% of the expected values are greater than 5; these conditions have not been met, and thus, the chi-square calculations are not valid.

### TB-IMID subjects had lower levels of IP-10 in response to TB1 and TB2 stimulation than TBI-NO IMID

**Figure 1:**
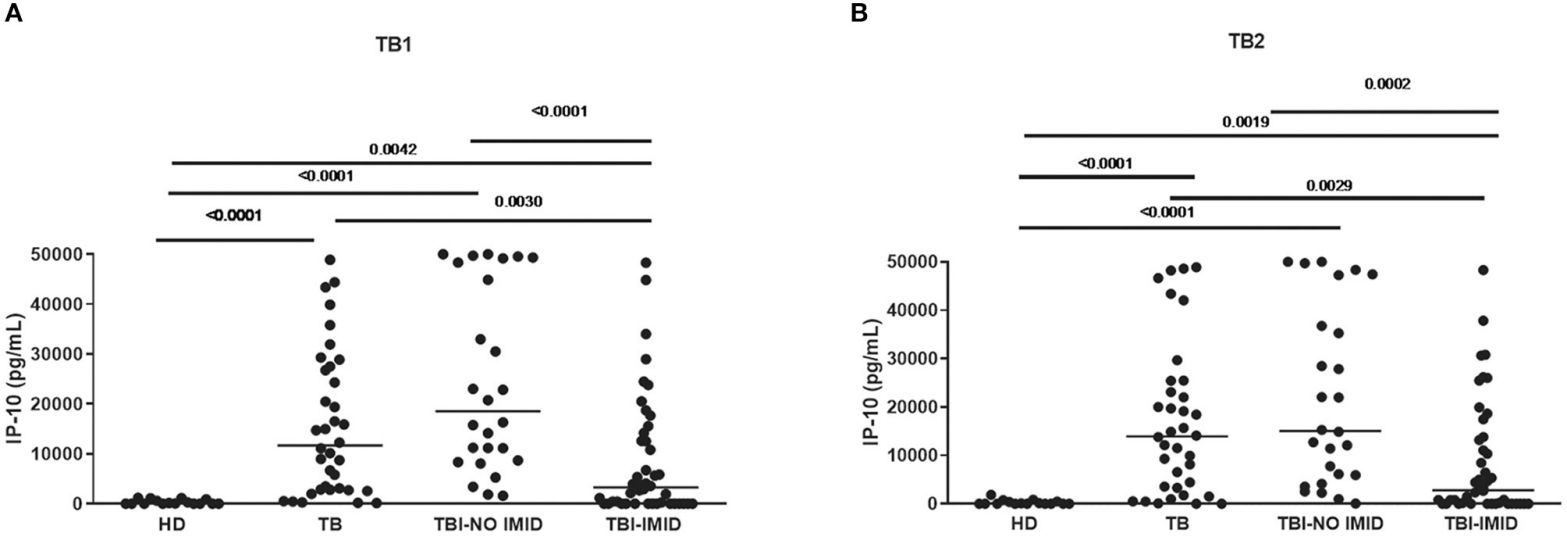
TBI-IMID patients had a lower level of IP-10 than TBI-NO IMID. **(A)** IP-10 levels in response to TB1 and **(B)** TB2 stimulation. ELISA was performed in plasma, and IP-10 was expressed as pg/mL. The horizontal lines represent the median; statistical analysis was performed using the Mann–Whitney test with Bonferroni correction, and the *p*-value was considered significant if < 0.008. IP-10, IFN-γ-inducible protein 10; TB, tuberculosis disease; TBI, TB infection; IMID, immune-mediated inflammatory disease; HD, healthy donor.

Compared with TBI-NO IMID patients, patients with TBI-IMID showed a significantly decreased IP-10 response to TB1 and TB2 stimulation (TB1: *p* < 0.0001; TB2: *p* = 0.0002) or TB disease (TB1: *p* = 0.003 and TB2: *p* = 0.0029) (Figures 1A, B). As previously described (49), we observed increased IP-10 levels in TB disease (TB1: *p* < 0.0001; TB2: *p* < 0.0001) and TBI (TB1: *p* < 0.0001; TB2: *p* < 0.0001) compared with HD; although the differences were less evident, similar results were obtained comparing TBI-IMID and HD (TB1: *p* = 0.0042; TB2: *p* = 0.0019) (Figures 1A, B).

Regarding the mitogen response, we did not observe any significant differences among groups (Supplementary Figure 2).

Among the TBI-IMID patients, no correlation between IP-10 and IFN-γ production with the number of lymphocytes was found (Supplementary Figure 3).

**Figure 2:**
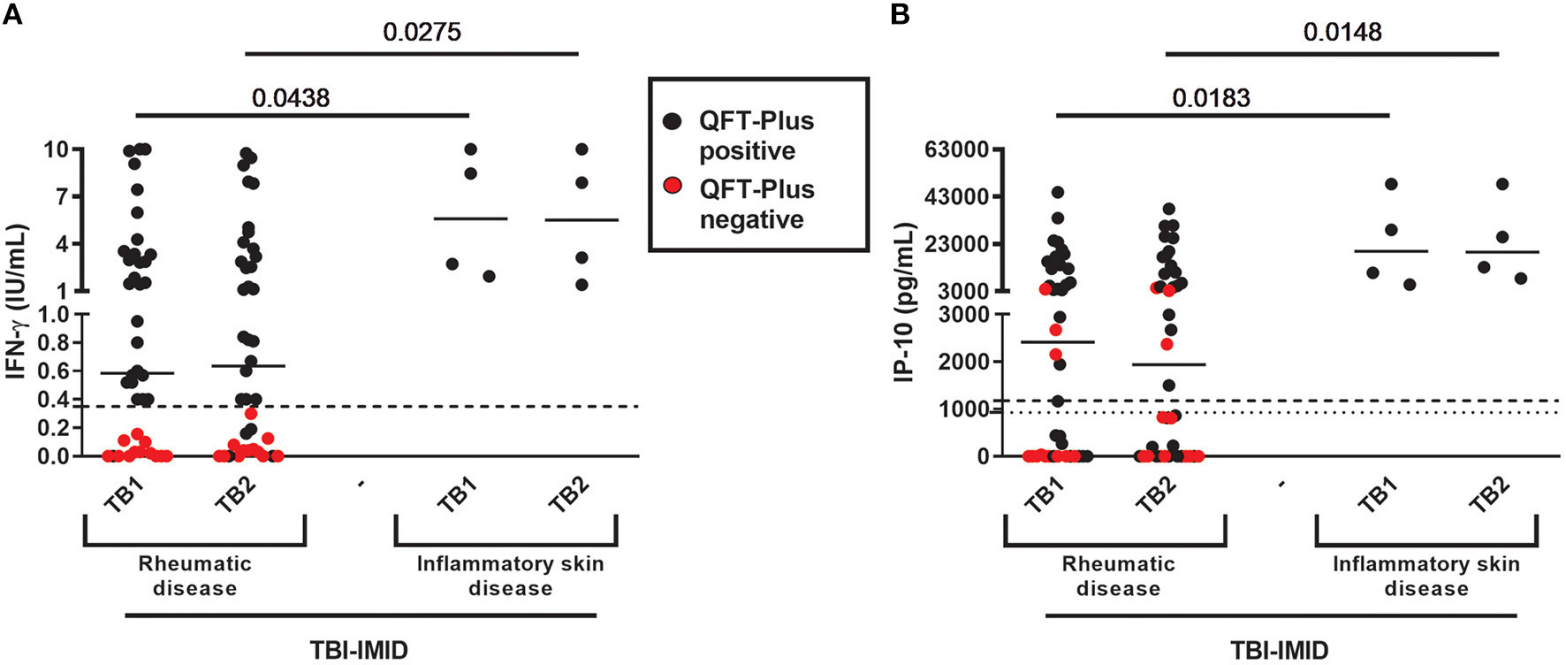
QFT-Plus and IP-10-based assay results in TBI-IMID stratified according to the type of IMID disease. **(A)** IFN-γ levels in response to TB1 and TB2 stimulation expressed as IU/mL; **(B)** IP-10 levels in response to TB1 and TB2 stimulation expressed as pg/mL. ELISA was performed in plasma. The horizontal lines represent the median; statistical analysis was performed using the Mann–Whitney test. IFN, interferon; IP-10, IFN-γ-inducible protein 10; QFT-Plus, QuantiFERON-TB-Plus; TBI, TB infection; IMID, immune-mediated inflammatory disease.

TBI-IMID QFT-Plus-negative belonged to the “rheumatoid arthritis” group (Table 3). According to these findings, the patients of the “rheumatoid arthritis” group had a lower median level of IFN-γ and IP-10 in response to TB1 and TB2 stimulation than group B (IFN-γ: *p* = 0.0438 and *p* = 0.0275, respectively; IP-10: *p* = 0.0183 and *p* = 0.0148, respectively) (Figure 2). Stratifying the TBI-IMID according to the type of IMID therapy (Supplementary Figures 1C, D), we did not find significant differences in terms of IP-10 production; however, the patients not in therapy showed a lower level of response to TB1 and TB2 stimulation than patients under therapy.

### Concordance between the IP-10-based assay and QFT-plus

Applying the IP-10 cut-off previously found to identify those with TB disease (TB1:1174 pg/Ml; TB2: 928.8 pg/mL) (49); we scored the results as positive and negative to TB1 or TB2 stimulation and we calculated the agreement between the IP-10 and QFT-Plus assays in the TBI-IMID group (Supplementary Table 1). We did not evaluate the agreement in the TBI-NO IMID group as TBI subjects were selected based on the positive score on QFT-Plus. In the TBI-IMID, we observed a moderate agreement in response to TB1 (k = 0.5), TB2 (k = 0.57), and TB1 or TB2 stimulation (k = 0.50). These results are in agreement with the concordance value obtained in the TB disease group in our previous study (49).

### Sensitivity of IP-10-based assay to detect TBI

As the TBI-NO IMID subjects were selected as IGRA-positive, it was unsuitable to evaluate the sensitivity in this group (Table 4). However, to compare the QFT-Plus results with those obtained by the IP-10-based assay, in Table 4, we reported the proportion of positivity of both tests. A similar percentage of positive scores was found in response to either TB1 or TB2 in TBI-NO IMID (IP-10: 100%, QFT-Plus 100%) (Table 4). Notably, three TBI-IMID patients who were negative on QFT-Plus were found positive on the IP-10-based test, whereas seven TBI-IMID patients who were positive on QFT-Plus were found negative on the IP-10-based test.

**Table 4:** Sensitivity of IP-10-based assay vs. QFT-plus in response to either TB1 or TB2.

**Tests**	**TBI-NO IMID *N =* 26**	**TBI-IMID *N =* 44**	**TBI-IMID QFT-positive *N =* 31**	**TBI-IMID QFT-negative *N =* 13**
**IP-10**				
Either TB1 or TB2 *N* (%)	26 (100)	27 (61)	24 (77)	3 (23)
TB1 *N* (%)	26 (100)	26 (59)	23 (74)	3 (23)
TB2 *N* (%)	25 (96)	25 (57)	22 (71)	3 (23)
**QFT-Plus**				
Either TB1 or TB2 *N* (%)	26 (100)	31 (70)	-	-
TB1 *N* (%)	26 (100)	31 (70)	-	-
TB2 *N* (%)	26 (100)	28 (64)	-	-
IP-10-based assay and QFT-plus	26 (100)	34 (77)	-	-

TB, tuberculosis; TBI, tuberculosis infection; HD, healthy donors; IMID, inflammatory-mediated immune disease; N, number; QFT-Plus, QuantiFERON-TB-Plus.

Differently, the TBI-IMID included both QFT-Plus positive and negative subjects; therefore, it was appropriate to evaluate the sensitivity of the TBI diagnosis for each test. QFT-Plus showed 70% sensitivity, whereas the IP-10-based assay reached a sensitivity of 61%. The combination of the two tests increased the sensitivity for TBI diagnosis up to 77%, as shown in Table 4.

### Analysis of QFT-plus results falling in the uncertain zone (0.2–0.7 IU/ml)

A value of IFN-γ ≥0.35 IU/mL defines the positivity to the QFT-Plus assay as specified by the manufacturer's instructions (59). Several studies have identified an uncertain zone (0.2–0.7 IU/mL) of the QFT-Plus results (31–33). Moreover, we have previously demonstrated that in TBI-IMID patients, a high proportion of IFN-γ results range in the uncertain zone (32).

Therefore, we stratified the results of TBI-IMID according to the uncertain zone of QFT-Plus results as follows: IFN-γ < 0.2 IU/mL; IFN-γ ranging in 0.2–0.07 IU/mL; IFN-γ >0.7 IU/mL (Supplementary Figures 4A, B).

Collectively among the TBI-IMID, 18% of TB1- (8 out of 44) and 14% of TB2-positive results (6 out of 44) were within the uncertain range (0.2–0.07 IU/mL). Furthermore, 52% of TB1- and TB2-positive results (23 out of 44) were out of the uncertain range (>0.07 IU/mL). In addition, 29.5% of TB1 and 34% of TB2 results (13 out of 44 and 15 out of 44) were in the certain negative result range (< 0.2 IU/mL). Among the TBI-IMID with a QFT-Plus negative result, the response was out of the uncertain range (< 0.2 IU/mL) for 13 out of 13 (100%) subjects in response to TB1 and 12 out of 13 (92%) subjects in response to TB2 (data not shown).

Ten TBI-IMID subjects had IP-10/IFN-γ discordant results (Supplementary Table 1; Supplementary Figure 4). Among them, 40% (TB1) and 30% (TB2) of IP-10-based assay results had corresponding IFN-γ values falling in the uncertain zone (Supplementary Figures 4C, D). The three patients who were positive on the IP-10-based test and who were negative on QFT-Plus had at least one IFN-γ value falling within the certain negative range (< 0.2 IU/mL) (Supplementary Figures 4C, D). However, 30% of the IP-10 results had corresponding IFN-γ values falling within the truly positive range of QFT-Plus (Supplementary Figures 4C, D).

### Analysis of the levels of several immune factors

To find alternative biomarkers for TBI-IMID diagnosis, we screened immune factors different from IP-10 or IFN-γ. In a subgroup of 81 subjects (22 TB, 25 TBI, 19 TBI-IMID, and 15 HD), we evaluated the plasma levels of several cytokines, chemokines, and growth factors after stimulation with TB1 and TB2 of QFT-Plus. The results obtained in response to TB1 and TB2 stimulation were strongly correlated (Table 5). Therefore, to simplify further analysis, we used the highest value obtained in response to TB1 or TB2 stimulation (TB-MAX). By using the gradient boosting machine (GBM) as a learning technique on TB-MAX results, we calculated a prediction model for the correct classification of the subjects with different TB statuses.

**Table 5:** Correlation of the TB1 with the TB2 results obtained by Luminex in all subjects analyzed.

**Analyte**	**Pearson's correlation coefficient**	***p***
IL-1β	0.61	0.000
IL-1rα	0.82	0.000
IL-2	0.95	0.000
IL-4	0.54	0.000
IL-5	0.71	0.000
IL-6	0.27	0.014
IL-7	0.61	0.000
IL-8	0.68	0.000
IL-9	0.66	0.000
IL-10	0.59	0.000
IL-12	0.69	0.000
IL-13	0.62	0.000
IL-15	0.74	0.000
IL-17A	0.45	0.000
Eotaxin	0.50	0.000
FGF-basic	0.47	0.000
G-CSF	0.80	0.000
GM-CSF	0.72	0.000
IFN-γ	0.94	0.000
IP-10	0.93	0.000
MCP-1	0.72	0.000
MIP-1α	0.83	0.000
PDGF	0.56	0.001
MIP-1β	0.72	0.000
RANTES	0.49	0.000
TNF-α	0.47	0.000
VEGF	0.64	0.000

FGF, fibroblast growth factor; G-CSF, granulocyte colony-stimulating factor; GM-CSF, granulocyte–macrophage colony-stimulating factor; IFN-γ, interferon- γ; IP-10, interferon-γ-inducible protein-10; MCP-1, monocyte chemoattractant protein-1; MIP-1α, macrophage inflammatory protein-1α; MIP1β, macrophage inflammatory protein-1β; PDGF, platelet-derived growth factor; RANTES, regulated on activation; normal T cell expressed and secreted; TNF-α, tumor necrosis factor-alpha; VEGF, vascular endothelial growth factor; IL, interleukin.

The GBM correctly classified 100% of TB disease, TBI, and HD subjects and 89.5% of TBI-IMID patients. Stratifying the TBI-IMID according to the QFT-Plus results, the GBM correctly classified 83% of TBI-IMID QFT-Plus-negative and 92% of TBI-IMID QFT-Plus-positive patients (Table 6).

**Table 6:** Gradient boosting machine results performed on the 27 analytes detected by Luminex.

		**Real patient diagnosis**					
	**Groups**	**HD** ***N** =* **15**	**TB** ***N** =* **22**	**TBI-NO IMID** ***N** =* **25**	**TBI-IMID** ***N** =* **19**	**TBI-IMID QFT-Plus positive** ***N** =* **13**	**TBI-IMID QFT-Plus negative** ***N** =* **6**
		***N*** **(%)**	***N*** **(%)**	***N*** **(%)**	***N*** **(%)**	***N*** **(%)**	***N*** **(%)**
GBM-based diagnosis	HD	15 (100)	0 (0)	0 (0)	1 (5.3)	0 (0)	1 (17)
	TB	0 (0)	22 (100)	0 (0)	0 (0)	0 (0)	0 (0)
	TBI-NO IMID	0 (0)	0 (0)	25 (100)	1 (5.2)	1 (8)	0 (0)
	TBI-IMID	0 (0)	0 (0)	0 (0)	17 (89.5)	12 (92)	5 (83)

TB, tuberculosis; TBI, tuberculosis infection; HD, healthy donors; IMID, inflammatory-mediated immune disease; N, number; QFT-Plus, QuantiFERON-TB-Plus; GBM, gradient boosting machine.

**Figure 3:**
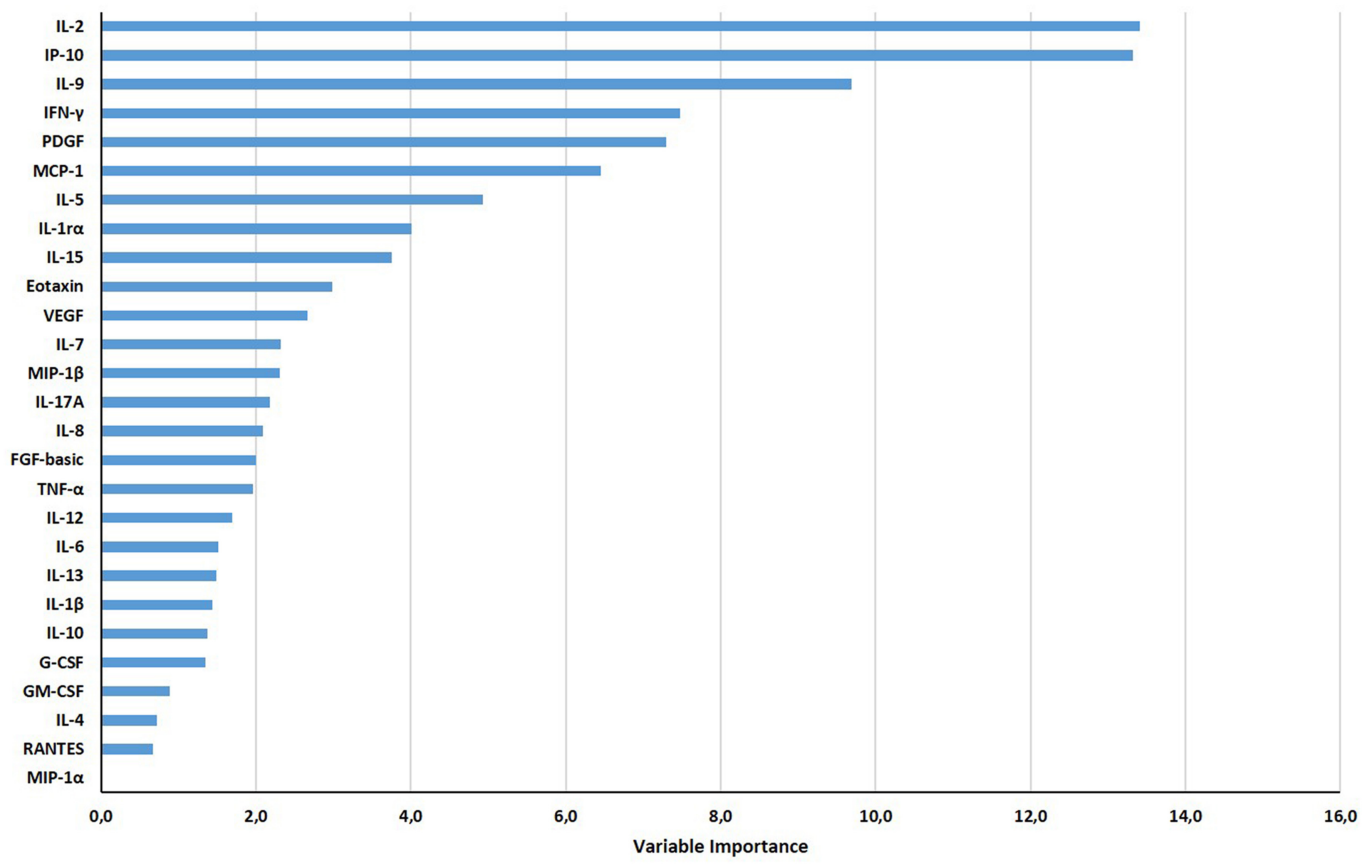
Gradient boosting machine (GBM) on Luminex data set of HD, TB, TBI-NO IMID, and TBI-IMID patients. The different soluble factors have been measured by Luminex assay in plasma collected after whole blood stimulation with TB1 and TB2. The graph ranks the individual variables based on their relative influence on the GBM; measuring the relative importance of each variable in training the model. The contribution of variables ranked from the top, the most important, to the bottom.

Based on the relative weight of the single variables on the training model construction (Figure 3), we identified IL-2, IP-10, and IL-9 as the most important analytes. The ROC analysis of TB disease, TBI, and TBI-IMID subjects vs. the HD group showed that these immune factors were highly associated with TB status (Table 7). Applying the cut-off identified by the comparison of TB disease with HD and TBI with HD, within the IMID population we did not gain a higher sensitivity for the TBI diagnosis than the QFT-Plus (Supplementary Table 2). Although we gained a higher sensitivity for the TBI diagnosis than the IP-10-based assay, the accuracy of the tests drastically decreased in TBI-IMID QFT-Plus negative (Supplementary Table 2). Moreover, stratifying the TBI-IMID according to the QFT-plus score vs. HD, the AUC (generated by the ROC analysis) of IL-2, IP-10, and IL-9 significantly decreased (Table 7).

**Table 7:** Receiver operating characteristic curves of selected analytes obtained by the Luminex assay for discriminating the different TB status from the HD.

**Analytes**	**GBM**	**TB disease**				**TBI-NO IMID**				**TBI-IMID**				**TBI-IMID QFT-Plus positive**				**TBI-IMID QFT-Plus negative**			
		**AUC**	**95%CI- inf**	**95%CI- sup**	***p***	**AUC**	**95%CI- inf**	**95%CI- sup**	***p***	**AUC**	**95%CI- inf**	**95%CI- sup**	***p***	**AUC**	**95%CI- inf**	**95%CI- sup**	***p***	**AUC**	**95%CI- inf**	**95%CI- sup**	***p***
IL-2	13.4	0.864	0.742	0.985	0.0002	1.000	1.000	1.000	< 0.0001	0.804	0.648	0.959	0.0025	0.980	0.936	1.000	< 0.0001	0.422	0.118	0.726	0.5858
IL-9	9.7	0.835	0.703	0.967	0.0005	0.920	0.833	1.000	< 0.0001	0.783	0.627	0.938	0.0045	0.836	0.683	0.989	0.0024	0.667	0.384	0.950	0.2429
IP-10	13.3	0.985	0.958	1.000	< 0.0001	0.989	0.969	1.000	< 0.0001	0.716	0.529	0.902	0.0329	0.882	0.741	1.000	0.0006	0.356	0.000	0.722	0.2129

TB, tuberculosis; TBI, tuberculosis infection; HD, healthy donors; IMID, inflammatory-mediated immune disease; N, number; QFT-Plus, QuantiFERON-TB-Plus; CI, confidence interval; sup, superior; inf, inferior; AUC, area under the curve.

To investigate the association of the increasing concentration of IL-2, IP-10, and IL-9 with TB status, we applied a multinomial logistic regression on these analytes comparing the different TB statuses with HD (Supplementary Table 3). We found that higher IP-10 levels were associated with higher increased RRR of being in the TB group (RRR: 1.02, 95%CI: 1.00–1.03, *p* = 0.022), whereas higher level of IL-2 was associated with both TB-NO IMID (RRR: 1.44, 95%CI: 1.01–2.04, *p* = 0.042) and TBI-NO IMID (RRR: 1.42, 95%CI: 1.00–2.01, *p* = 0.051). In this final comparison, the result was close to significance.

These results supported the strict association between the analytes previously identified with the GBM model and the different stages of TB. As expected, the association of IL-2 with TBI-IMID was close to significance, highlighting the struggle to find alternative and valid biomarkers in this particular cohort of patients.

## Discussion

In this study, we evaluated in the QFT-Plus platform the possibility of finding biomarkers alternative to IFN-γ for TBI diagnosis in a cohort of individuals with IMID candidates to biological therapy.

In particular, the cohort of TBI-IMID patients included individuals who were positive on QFT-Plus but also subjects who were negative on QFT-Plus but with radiological evidence of TB exposure such as fibrosis in the upper lungs lobes, persistent calcification or persistent mass-like opacities (60), and with reported previous exposure to TB cases in the past. This TBI-IMID cohort, who were QFT-Plus negative, represents an important population to study alternative immunological biomarkers for TBI diagnosis.

Differently from previous studies (32) based on the detection of IFN-γ to QFT-Plus, in the present report the IP-10 production in response to the mitogen stimulation was not affected by the IMID status. However, the IMID status had a negative impact on the TB1 and TB2 response as already observed in a larger cohort of TBI-IMID and TBI-NO IMID (32). To note that the IMID patients with articular involvement had a more compromised IP-10 and IFN-γ response compared with subjects with skin inflammatory diseases. According to these findings, the TBI-IMID subjects who were QFT-Plus negative had diseases such as rheumatoid arthritis, polymyalgia rheumatica, and psoriatic arthritis. Moreover, the number of lymphocytes did not affect either IP-10 or IFN-γ response to QFT-plus as already demonstrated in the past only for the IFN-γ response (32).

Altogether, these data strongly suggest a similar modulation of IFN-γ and IP-10 response to QFT-Plus in TBI-IMID individuals and reflect a high likelihood of IMID subjects with an articular involvement to have a negative QFT-Plus score. Interestingly, 46% of the TBI-IMID subjects were QFT-Plus negative and were treatment free at the time of the enrolment. The analysis of the results according to the “IFN-γ uncertain range” confirmed that some of the TBI-IMID had IFN-γ values falling in the uncertain range as already demonstrated (32).

We did not find a direct correlation between the values considered “uncertain results” of QFT-Plus and the IP-10 values as only 30–40% of discordant IP-10-based assay/QFT-Plus results had an IFN-γ value within the uncertain range. Moreover, the IP-10-based assay allowed to regain 3 TBI-IMID subjects with truly negative IFN-γ results (< 0.2) obtained by QFT-Plus (28, 32, 61). According to the QFT-Plus results, these patients were not diagnosed with TBI, and based on this score, TB preventive therapy was not indicated. As these patients had radiological findings suggestive of TBI, we may speculate that the IP-10-based assay correctly diagnosed TBI. Nevertheless, the IP-10-based assay failed to diagnose 10 subjects who were positive on QFT-Plus. All these results suggest that the IP-10-based assay alone cannot help solve the doubtful QFT-Plus results. However, the simultaneous use of IP-10 and QFT-Plus assays may improve the sensitivity for TBI diagnosis in TBI-IMID.

By applying a gradient boosting machine methodology, we explored the possibility of using alternative biomarkers to correctly diagnose TBI in the IMID population and we reached 89% sensitivity for diagnosis of TBI. By this approach, we used all the 27 analytes evaluated by the multiplex assay, to develop a prediction model for the correct classification of TBI-IMID subjects. Usually, the ROC analysis is run on patients with a certain disease vs. a control population. In our study, the TB disease population was certainly Mtb-infected and the HD population was certainly uninfected. As Mtb cannot be isolated in the TBI subjects by definition, in our study we used as a surrogate of true Mtb infection the positive QFT-Plus used for the definition of TBI (10). Based on this assumption, we directly analyzed the TBI-IMID group vs. the HD to find alternative biomarkers. A limitation of this method is the use of a large data set. For this reason, this kind of approach is exploratory by definition (62). By the GBM ranking of variables, we identified three immune factors (IL-2, IP-10, and IL-9) with the highest relative importance and the highest AUC to discriminate the different TB status from the HD control group. Nevertheless, none of the immune factors discriminated TBI-IMID subjects from HD with an accuracy higher than QFT-Plus. To note that by GBM the sensitivity for the TBI diagnosis of IMID patients was higher than the IP-10-based assay; however, the accuracy of the tests decreased in TBI-IMID QFT-Plus negative. Finally, we did not find biomarkers alternative to IFN-γ to diagnose TBI within the IMID population who were negative on QFT-Plus and were diagnosed as TBI by using radiological tools, even after analyzing 27 different biomarkers.

Limitations of this prospective study are the relatively low number of patients enrolled, mainly within the TBI-IMID group with a negative QFT-Plus score, the variety of the pathologies within the IMID considered, and the different regimens of therapy for the IMID. However, this study focused attention on a difficult clinical issue as the diagnosis of TBI in a fragile category, such as the IMID patients. Moreover, the variety of therapies mirrors the clinical scenario of these patients. Alternative biomarkers in QFT-Plus negative TBI-IMID subjects are missing. Considering the possible risk of developing TB disease in this population, a negative QFT-Plus result should be always carefully considered and a chest X-ray evaluation should be performed, as already recommended by several guidelines (63), to ensure TBI diagnosis and eventually to provide TB preventive treatment. It has been demonstrated that long-term stimulation with specific Mtb antigens may enhance the Mtb-specific response (64, 65). As the TBI-IMID subjects had probably remote TB exposure, future studies are needed to evaluate the memory response of TBI subjects with a radiological diagnosis and to develop alternative strategies for immune diagnosis of TBI. Based on the trouble of detecting a specific immune response in this IMID population, another approach could be a research assay based on the Mtb DNA evaluation in the peripheral blood (66, 67).

In conclusion, in this study, we found a moderate agreement between the IP-10-based assay and QFT-Plus in TBI-IMID, with an increased sensitivity for TBI diagnosis up to 77% when tests were combined. The evaluation of alternative biomarkers such as IL-2, IP-10, and IL-9 did not allow an improvement of sensitivity for the TBI diagnosis in the IMID population; moreover, we demonstrated a decreased accuracy as evaluated by the analysis of the AUC of these factors in TBI-IMID with a radiological diagnosis and a negative score to the QFT-Plus. Although preliminary, these data highlight the need to develop alternative strategies for TBI immune diagnosis in particular in TBI-IMID patients with radiological diagnosis. These results may help in the management of tuberculosis in patients taking lifelong immune-suppressive drugs.

## Data availability statement

The datasets presented in this study can be found in online repositories. The names of the repository/repositories and accession number(s) can be found at: The data sets generated during and/or analyzed during the current study are available in our institutional repository after request at rawdata.inmi.it.

## Ethics statement

The studies involving humans were approved by the Comitato Etico Lazzaro Spallanzani. The studies were conducted in accordance with the local legislation and institutional requirements. The participants provided their written informed consent to participate in this study.

## Author contributions

EP: Funding acquisition, Investigation, Data curation, Formal analysis, Project administration, Writing—original draft. LP: Data curation, Formal analysis, Investigation, Methodology, Writing—review & editing. SN-F: Methodology, Writing—review & editing. AN: Writing—review & editing, Data curation, Formal analysis. VV: Writing—review & editing, Methodology. GC: Writing—review & editing, Data curation. FC: Writing—review & editing. GG: Writing—review & editing. FP: Writing—review & editing. DG: Writing—review & editing, Funding acquisition, Investigation, Supervision.

## Supplementary material

Supplementary Figure 1 QFT-Plus and I0–10 assay results in TBI-IMID stratified according to the type of IMID therapy. **(A, B)** IFN-γ levels in response to TB1 and TB2 stimulation expressed as IU/mL; **(C, D)** IP-10 levels in response to TB1 and TB2 stimulation expressed as pg/mL. ELISA was performed in plasma. The horizontal lines represent the median; statistical analysis was performed using the Mann-Whitney test. IFN, interferon; IP-10, IFN-γ inducible protein 10; TBI, TB infection; IMID, immune-mediated inflammatory disease; QFT-Plus, QuantiFERON-TB-Plus; combination: immune suppressive drugs ± corticosteroids ± biologics ± non-steroidal anti-inflammatory drugs. One drug treatment: immune suppressive drugs or corticosteroids or non-steroidal anti-inflammatory drugs. Only biologic: 2 patients under anti-TNF-a, 2 patients under anti-IL-6 receptor.

Supplementary Figure 2 TBI-IMID has a mitogen response of IP-10-based assay similar to TBI-NO IMID. ELISA was performed in plasma and the IP-10 level is expressed as pg/mL. The horizontal lines represent the median; statistical analysis was performed using the Mann–Whitney test. IP-10, IFN-γ inducible protein 10; TB, tuberculosis; TBI, TB infection; IMID, immune-mediated inflammatory disease; HD, healthy donor.

Supplementary Figure 3 Correlation of IP-10 and IFN-γ levels with the number of lymphocytes in TBI-IMID. ELISA was performed in plasma and IP-10 level is expressed as pg/mL and IFN-γ in IU/mL. Statistical analysis was performed using the Spearman correlation test. The lymphocyte number was not available for 7 patients; therefore, the test has been performed on 37 subjects. IFN, interferon; IP-10, IFN-γ inducible protein 10.

Supplementary Figure 4 QFT-Plus and IP-10-based assay results in TBI-IMID stratified according to the uncertain range of QFT-Plus assay. **(A, B)** IFN-γ levels in response to TB1 and TB2 stimulation expressed as IU/mL; results have been stratified according to the uncertain range of QFT-Plus; **(C, D)** IP-10 levels in response to TB1 and TB2 stimulation expressed as pg/mL; IP-10 results have been stratified according to the uncertain range distribution of correspondent IFN-γ values. ELISA was performed in plasma. The horizontal lines represent the median; statistical analysis was performed using the Mann–Whitney test. IFN, interferon; IP-10, IFN-γ inducible protein 10; TBI, TB infection; IMID, immune-mediated inflammatory disease. Colored plots represent the combination of QFT-Plus and IP-10 results scores. Definition of IFN-γ range: values ≤ 0.2 = negative; < 0.2 > 07 = uncertain; ≥0.7 positive.

Supplementary Table 1 Concordance between IP-10-based assay and QFT-Plus in TBI-IMID patients. TB, tuberculosis; TBI, tuberculosis infection; HD, Healthy donors; IMID, inflammatory mediated immune disease; N, number; IP-10, Interferon-γ Inducible Protein 10; QFT-Plus, QuantiFERON-TB-Plus; ^*^Chi-square test; na, not applicable; k, Cohen's kappa coefficient.

Supplementary Table 2 ROC analysis generated on IL-2, IL-9, IP-10 results in subjects with different TB status. TB, tuberculosis; TBI, tuberculosis infection; HD, Healthy donors; IMID, inflammatory mediated immune disease; N, number; QFT-Plus, QuantiFERON-TB-Plus; CI, confidence interval; sup, superior; inf, inferior; AUC, area under the curve.

Supplementary Table 3 Relative risk ratio of IL-2, IL-9 and IP-10 within TB, TBI-NO IMID and TBI-IMID. RRR, relative risk ratio; CI, confidence interval; TB, tuberculosis; TBI, tuberculosis infection; IMID, inflammatory mediated immune disease; HD, healthy donor; ^*^Association of increasing concentration (ten-unit increase) of IL-2, IL-9 and IP-10 within TB, TBI-NO IMID and TBI-IMID compared to HD by applying multinomial logistic regression.
