# Supplementary figures and images for: Alternative biomarkers of tuberculosis infection in patients with immune-mediated inflammatory diseases
Source: Front Med (Lausanne). 2023 Nov 23;10:1271632. doi: 10.3389/fmed.2023.1271632 (PMC10704032; doi:10.3389/fmed.2023.1271632)

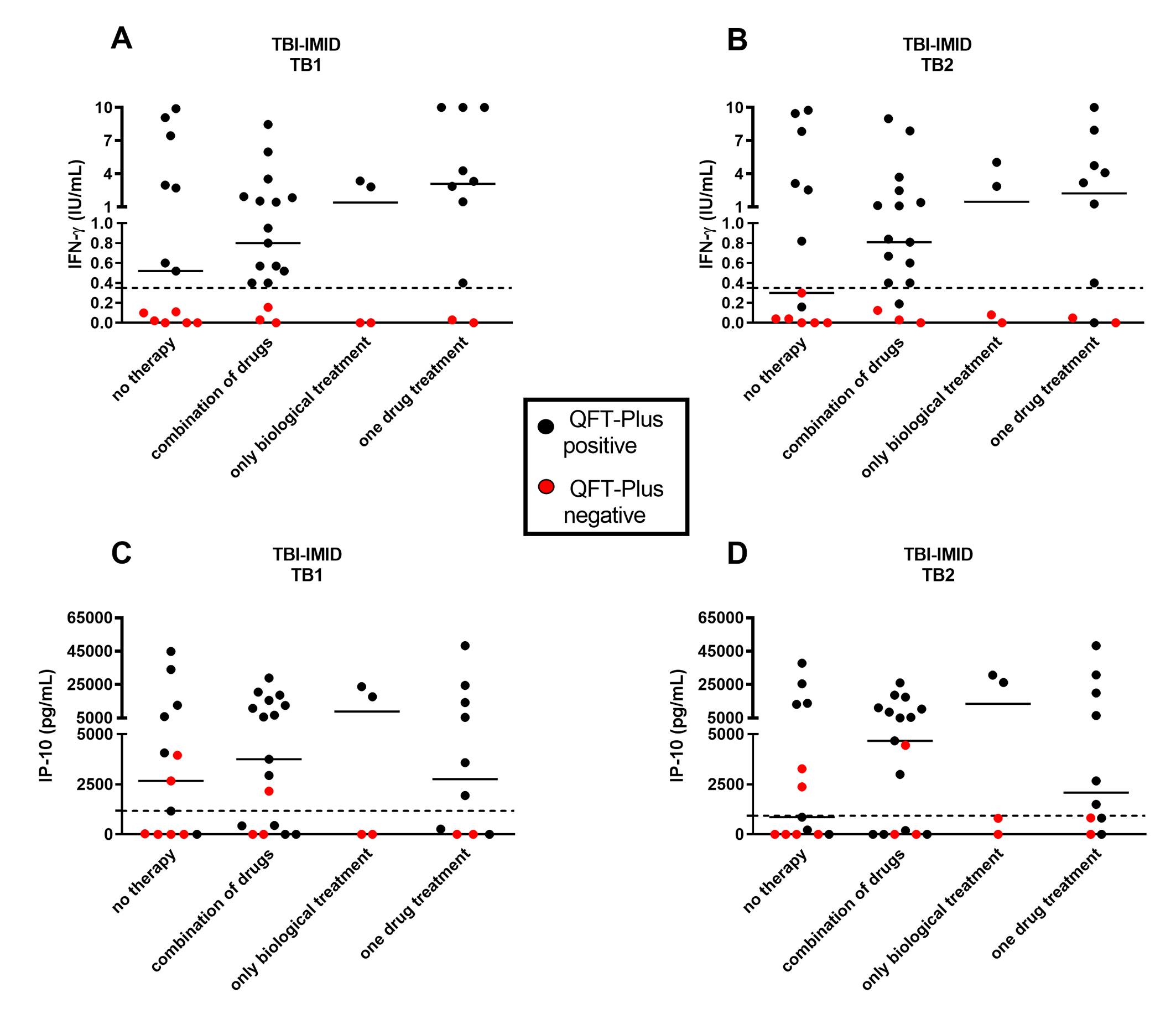

Supplement: Supplementary Figure 1 — QFT-Plus and I0–10 assay results in TBI-IMID stratified according to the type of IMID therapy. (A, B) IFN-γ levels in response to TB1 and TB2 stimulation expressed as IU/mL; (C, D) IP-10 levels in response to TB1 and TB2 stimulation expressed as pg/mL. ELISA was performed in plasma. The horizontal lines represent the median; statistical analysis was performed using the Mann-Whitney test. IFN, interferon; IP-10, IFN-γ inducible protein 10; TBI, TB infection; IMID, immune-mediated inflammatory disease; QFT-Plus, QuantiFERON-TB-Plus; combination: immune suppressive drugs ± corticosteroids ± biologics ± non-steroidal anti-inflammatory drugs. One drug treatment: immune suppressive drugs or corticosteroids or non-steroidal anti-inflammatory drugs. Only biologic: 2 patients under anti-TNF-a, 2 patients under anti-IL-6 receptor. [file Image_1.TIF]

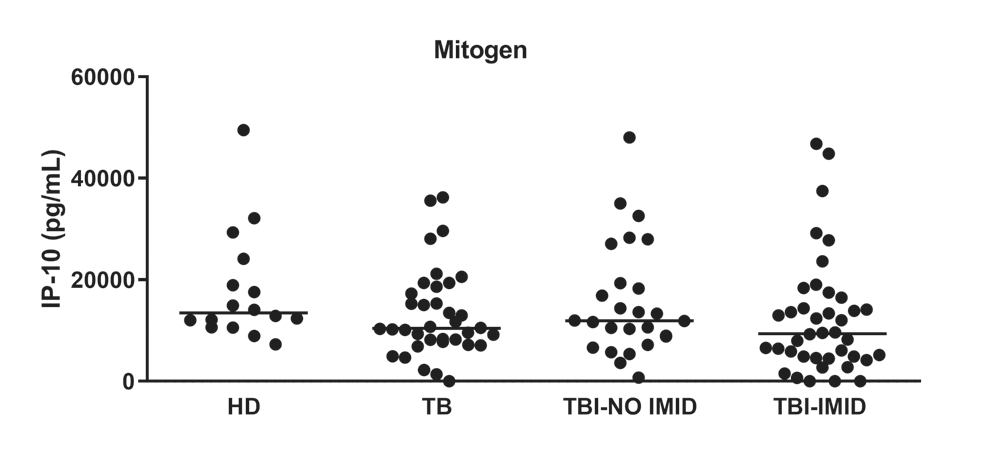

Supplement: Supplementary Figure 2 — TBI-IMID has a mitogen response of IP-10-based assay similar to TBI-NO IMID. ELISA was performed in plasma and the IP-10 level is expressed as pg/mL. The horizontal lines represent the median; statistical analysis was performed using the Mann–Whitney test. IP-10, IFN-γ inducible protein 10; TB, tuberculosis; TBI, TB infection; IMID, immune-mediated inflammatory disease; HD, healthy donor. [file Image_2.TIF]

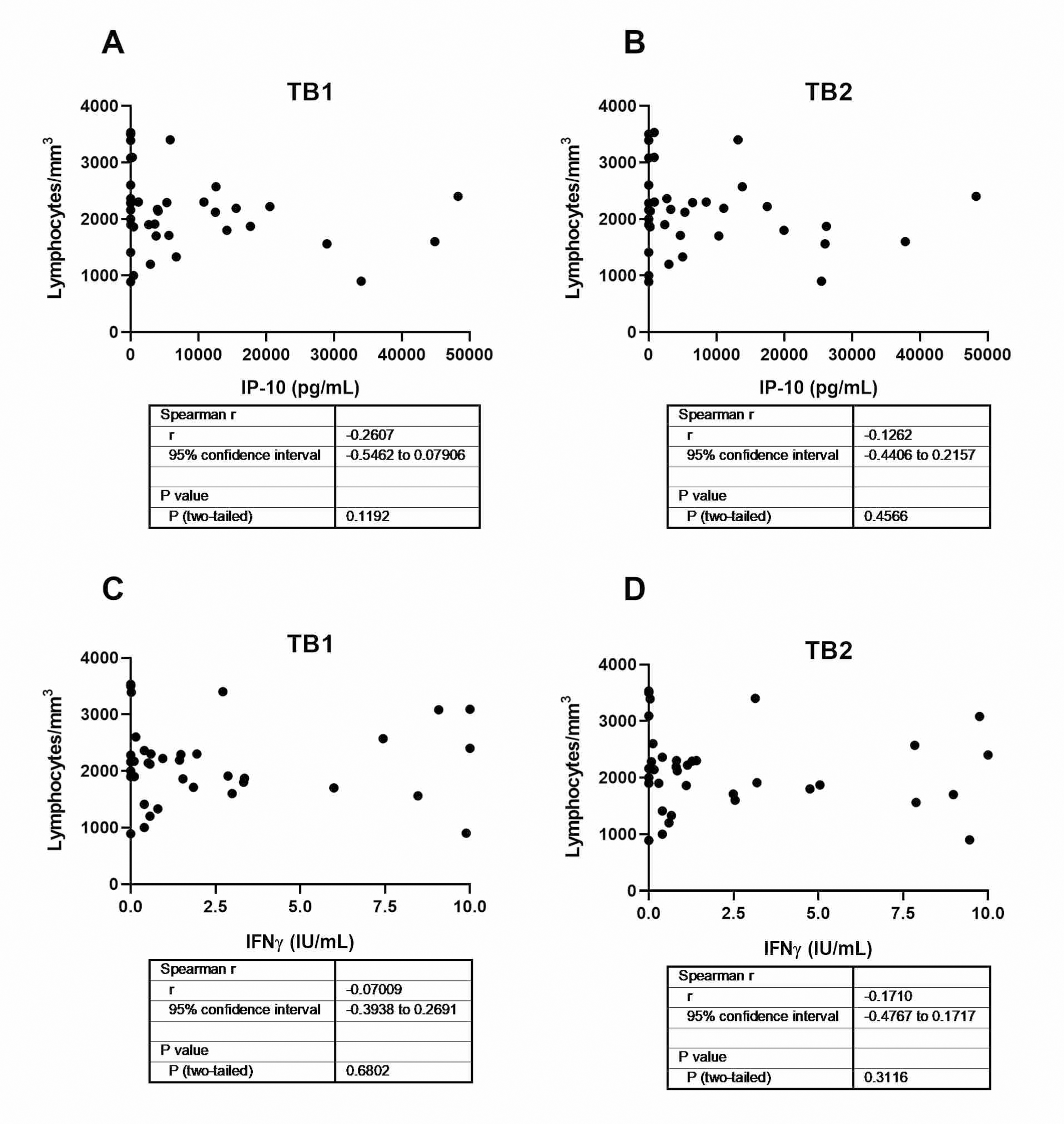

Supplement: Supplementary Figure 3 — Correlation of IP-10 and IFN-γ levels with the number of lymphocytes in TBI-IMID. ELISA was performed in plasma and IP-10 level is expressed as pg/mL and IFN-γ in IU/mL. Statistical analysis was performed using the Spearman correlation test. The lymphocyte number was not available for 7 patients; therefore, the test has been performed on 37 subjects. IFN, interferon; IP-10, IFN-γ inducible protein 10. [file Image_3.TIF]

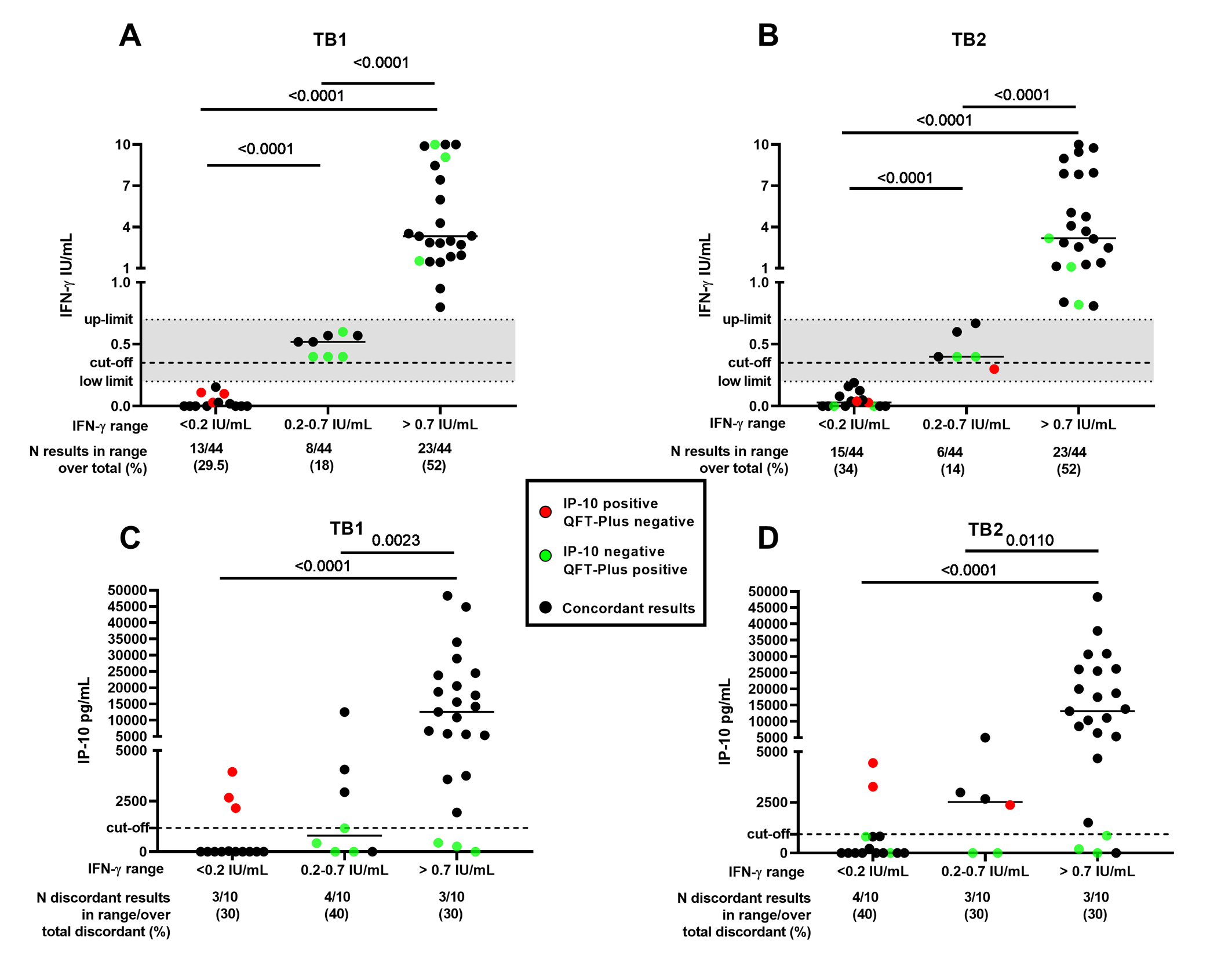

Supplement: Supplementary Figure 4 — QFT-Plus and IP-10-based assay results in TBI-IMID stratified according to the uncertain range of QFT-Plus assay. (A, B) IFN-γ levels in response to TB1 and TB2 stimulation expressed as IU/mL; results have been stratified according to the uncertain range of QFT-Plus; (C, D) IP-10 levels in response to TB1 and TB2 stimulation expressed as pg/mL; IP-10 results have been stratified according to the uncertain range distribution of correspondent IFN-γ values. ELISA was performed in plasma. The horizontal lines represent the median; statistical analysis was performed using the Mann–Whitney test. IFN, interferon; IP-10, IFN-γ inducible protein 10; TBI, TB infection; IMID, immune-mediated inflammatory disease. Colored plots represent the combination of QFT-Plus and IP-10 results scores. Definition of IFN-γ range: values ≤ 0.2 = negative; < 0.2 > 07 = uncertain; ≥0.7 positive. [file Image_4.TIF]
