# Supplementary material for: Alternative biomarkers of tuberculosis infection in patients with immune-mediated inflammatory diseases
Source: Front Med (Lausanne). 2023 Nov 23;10:1271632. doi: 10.3389/fmed.2023.1271632 (PMC10704032; doi:10.3389/fmed.2023.1271632)
Supplement: Supplementary Table 1 — Concordance between IP-10-based assay and QFT-Plus in TBI-IMID patients. TB, tuberculosis; TBI, tuberculosis infection; HD, Healthy donors; IMID, inflammatory mediated immune disease; N, number; IP-10, Interferon-γ Inducible Protein 10; QFT-Plus, QuantiFERON-TB-Plus; *Chi-square test; na, not applicable; k, Cohen's kappa coefficient. [file Table_1.pdf]

Supplementary table 1. Concordance between IP-10-based assay and QFT-Plus in TBI-IMID patients

	TB1		TB2		TBI1 OR TB2	
	TBI NO-IMID	TBI-IMID	TBI NO-IMID	TBI-IMID	TBI NO-IMID	TBI-IMID
**N**	26	44	26	44	26	44
**IP-10 positive/QFT-Plus positive N (%)**	26 (100)	24 (54)	25 (96)	22 (50)	26 (100)	24 (54)
**IP-10 positive/QFT-Plus negative N (%)**	0 (0)	3 (7)	0 (0)	3 (7)	0 (100)	3 (7)
**IP-10 negative/QFT-Plus positive N (%)**	0 (0)	7 (16)	1 (4)	6 (14)	0 (100)	7 (16)
**IP-10 negative/QFT-Plus negative N (%)**	0 (0)	10 (23)	0 (0)	13 (29)	0 (100)	10 (23)
**K (CI)**	na	0.50 (0.23-0.76)	na	0.57 (0.33-0.82)	na	0.50 (0.23-0.76)
**p ***	na	0.0007	na	0.0001	na	0.0007

Footnotes: TB: tuberculosis; TBI: tuberculosis infection; HD: Healthy donors; IMID: inflammatory mediated immune disease; N: number; IP-10: Interferon-γ Inducible Protein 10; QFT-Plus: QuantiFERON-TB-Plus *Chi-square test; na = not applicable; k = Cohen's kappa coefficien

Supplementary table 2. ROC analysis generated on IL-2, IL-9, IP-10 results in subjects with different TB status

	Cut-off generated with ROC analysis of TB disease versus HD	TBI-IMID diagnosis based on selected cut-off		Cut-off generated with ROC analysis of TBI-NO IMID versus HD	TBI-IMID diagnosis based on selected cut-off	
**Immune factors**		**Sensitivity**			**Sensitivity**	
**IL-2**	47.44	**All**	68.4	47.44	**All**	68.4
		**Within QFT-Plus positive**	92.3		**Within QFT-Plus positive**	92.3
		**Within QFT-Plus negative**	16.7		**Within QFT-Plus negative**	16.7
**IL-9**	31.36	**All**	36.8	37.24	**All**	31.6
		**Within QFT-Plus positive**	46.2		**Within QFT-Plus positive**	38.5
		**Within QFT-Plus negative**	16.7		**Within QFT-Plus negative**	16.7
**IP-10**	453.08	**All**	68.4	1125.88	**All**	52.6
		**Within QFT-Plus positive**	84.6		**Within QFT-Plus positive**	69.2
		**Within QFT-Plus negative**	33.3		**Within QFT-Plus negative**	16.7

Footnotes: TB: tuberculosis; TBI: tuberculosis infection; HD: Healthy donors; IMID: inflammatory mediated immune disease; N: number; QFT-Plus: QuantiFERON-TB-Plus; CI: confidence interval; sup: superior; inf: inferior; AUC: area under the curve

Supplementary table 3: relative risk ratio of IL-2, IL-9 and IP-10 within TB, TBI-NO IMID and TBI-IMID

	Analytes	RRR*	95 %CI	p
**TB vs HD**	IL-2	1.14	0.79-1.64	0.492
	IL-9	1.67	0.81-3.44	0.163
	IP-10	1.02	1.00-1.03	0.022
**TBI-NO IMID vs HD**	IL-2	1.44	1.01-2.04	0.042
	IL-9	1.85	0.90-3.80	0.096
	IP-10	1.01	1.00-1.03	0.132
**TBI-IMID vs HD**	IL-2	1.42	1.00-2.01	0.051
	IL-9	1.68	0.82-3.45	0.159
	IP-10	1.01	1.00-1.03	0.114

Footnotes: RRR: relative risk ratio; CI confidence interval, TB: tuberculosis; TBI: tuberculosis infection; IMID: inflammatory mediated immune disease; HD: healthy donor; *association of increasing concentration (ten-unit increase) of IL-2, IL-9 and IP-10 within TB, TBI-NO IMID and TBI-IMID compared to HD by applying multinomial logistic regression.
